# Secondary glioblastoma after treatment of intracranial germinoma - would radiation-only therapy still be safe? Case report

**DOI:** 10.1186/s12885-018-5073-3

**Published:** 2018-11-16

**Authors:** Kihwan Hwang, Kyu Sang Lee, Gheeyoung Choe, Byung-Gyu Cho, Chae-Yong Kim

**Affiliations:** 1 Department of Neurosurgery, Seoul National University Bundang Hospital, 82, Gumi-ro 173 Beon-gil, Bundang-gu, Seongnam-si, Gyeonggi-do 13620 Republic of Korea; 2 Department of Pathology, Seoul National University Bundang Hospital, 82, Gumi-ro 173 Beon-gil, Bundang-gu, Seongnam-si, Gyeonggi-do 13620 Republic of Korea; 3 Department of Neurosurgery, Korean Armed Forces Capital Hospital, Seongnam-si, South Korea; 4 Seoul National University College of Medicine, Seoul, South Korea

**Keywords:** Germinoma, Radiotherapy, Secondary malignancy, Glioblastoma

## Abstract

**Back ground:**

Intracranial germinomas are one of the most radiosensitive tumors and are curable by radiotherapy (RT) alone. RT-only therapy without chemotherapy is effective. But, as patients with germinoma can expect long-term survival, the adverse effects of RT and late sequelae in survivors are of most concern. So, recently, standard treatment protocol of combination with chemotherapy and reduced dose of RT could be widely acceptable.

**Case presentation:**

We report a patient with germinoma who developed RT-induced glioblastoma. He was diagnosed as biopsy-proven germinoma at the age of 12. Postoperatively, he underwent RT alone without chemotherapy and remained free of tumor without recurrence during long-term follow up. However, after almost 20 year, he developed RT-induced glioblastoma.

**Conclusions:**

Although RT has the highest priority among treatments on intracranial germinomas, RT-only therapy with full dose for germinoma can have delayed severe complications. So, chemotherapy prior to reduced dose RT is more desirable.

## Background

Intracranial germinomas are one of the most radiosensitive tumors and are curable by radiotherapy (RT) alone [1–3]. As patients with intracranial germinoma can expect long-term survival, the adverse effects of RT and late sequelae in survivors are inevitable. RT-induced sencodary malignancy is one of those sequelae. To the best of our knowledge, only a few cases of radiation-induced secondary tumors have been reported in germ cell tumors (GCT), especially in pure germinomas [4–6]. Herein, we describe follow-up results of a germinoma case which developed therapy-associated secondary tumor.

Intracranial germinoma have favorable cure rates. Acharya et al. reported that overall survival at 20 and 30 years for GCT was 84.1 and 61.9%, respectively, and the cumulative incidence of subsequent malignancy after treatment of germinoma was 6.0% at 25 years [7]. As, late sequelae affect long-term survival, treatment protocol of combination with chemotherapy and reduced dose of RT is widely acceptable, recently [8–14]. Our case would support the change for treatment protocol of combined chemotherapy and RT.

## Case presentation

**Fig. 1:**
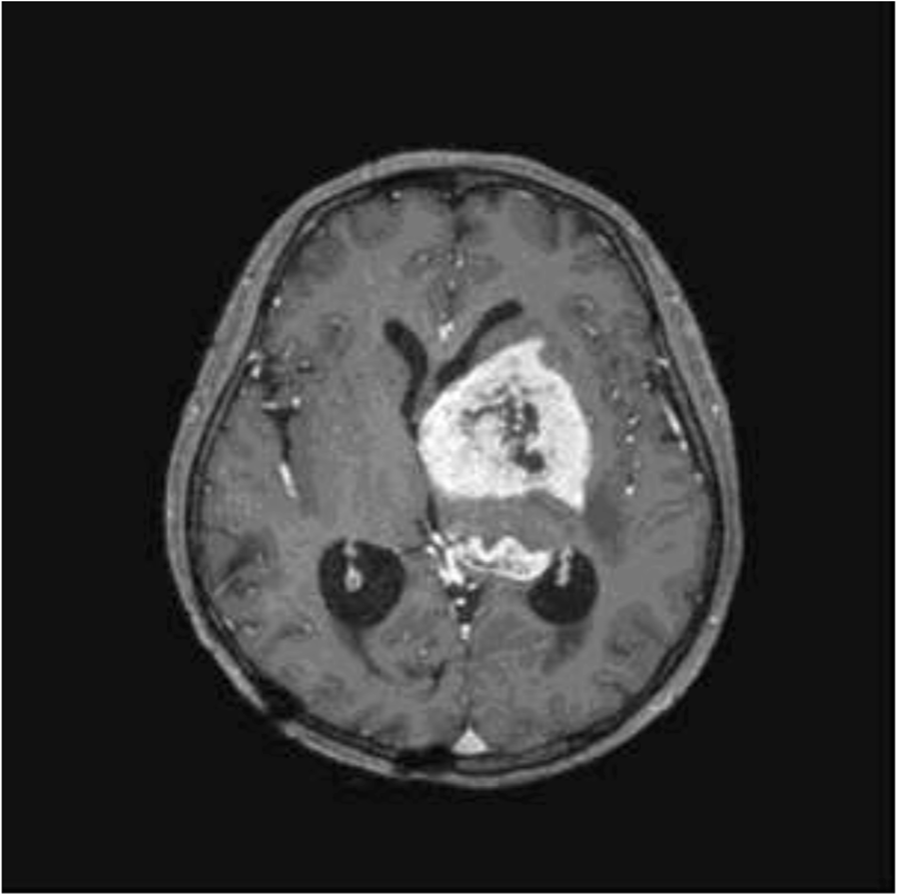
MRI demonstrated a tumor as a gadolinium-enhanced lesion in the left basal ganglia with extension to crus cerebri and thalamus

A 33-year-old man presented with right facial palsy and right hand fine motor dysfunction for over previous 3 months. MRI revealed a gadolinium-enhanced mass lesion in left basal ganglia with extension to crus cerebri and left thalamus (Fig. 1). Stereotactic biopsy was performed and the lesion was identified as glioblastoma, IDH-wild type.

The past medical history revealed the patient was once diagnosed with pineal mass for almost 20 years ago in 1995, when he was a 12-year-old boy. At that time, he underwent total removal of tumor via the right occipital transtentorial approach and biopsy identified germinoma. Postoperatively, he received craniospinal irradiation 24Gy and whole-brain radiotherapy 36Gy, that is, tumor bed total 50.4Gy. He was free from tumor recurrence or secondary tumor until 2006, when he had the last follow-up MRI. After then, he had no MRI follow-ups until the newly-developed symptoms occur in January 2015.

**Fig. 2:**
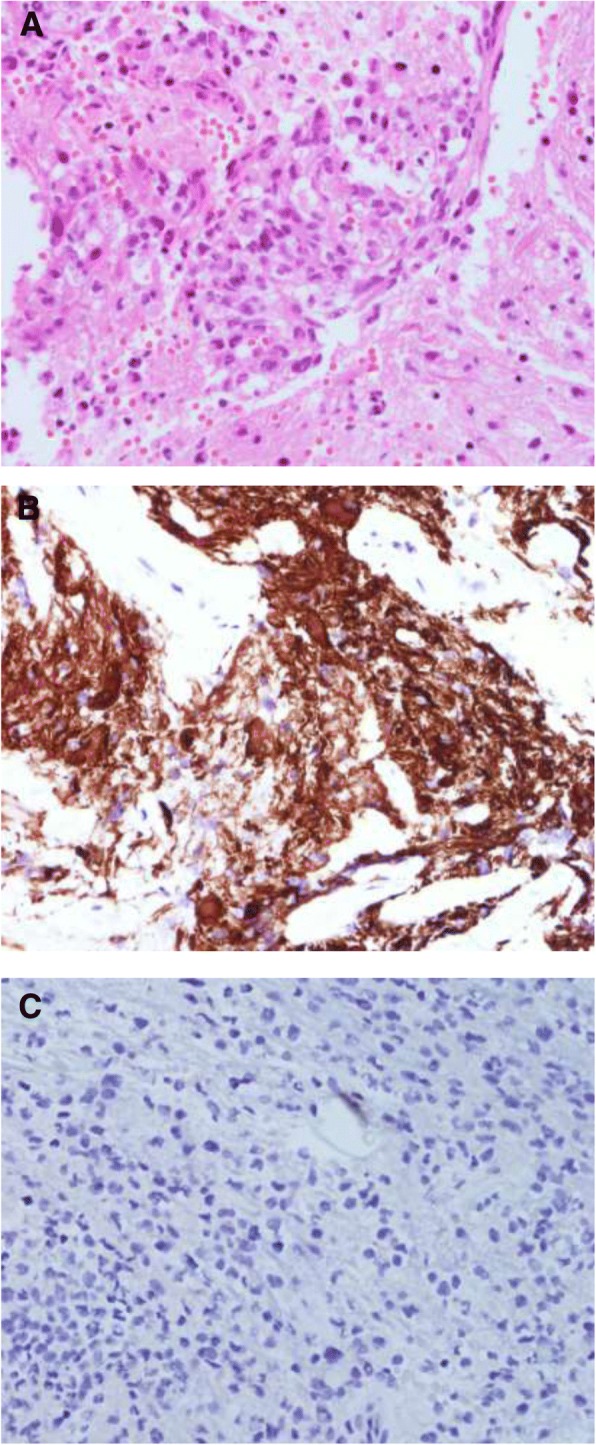
Histopathological examination of the surgical specimen. (**a**) Hematoxylin and eosin stain (× 400) revealed a poorly differentiated malignant tumor. Immunohistochemistry stains (× 400) were (**b**) positive for GFAP and (**c**) negative for IDH-1

Although we thought that this was evidence of tumor recurrence, biopsy identified glioblastoma, and we suspected that it was a therapy-associated tumor (Fig. 2). He received concurrent chemoradiotherapy with temozolomide. As tumor progression was identified during follow-up, he underwent bevacizumab/irinotecan and metronomic temozolomide consecutively till 15 months after the biopsy. But, the tumor progressed and he died in May 2016.

## Discussion and conclusions

The authors reported secondary malignancy from GCT that occurred after cranial irradiation to emphasize careful monitoring for long-term survival for GCT. The accepted criteria to diagnose a RT induced brain tumor are well defined as follows: 1) the tumor must appear within the irradiated field; 2) the tumor was not present prior to the RT; 3) a sufficient latency period must elapse between irradiation and appearance of the tumor (usually > 5 years); 4) the radiation induced tumor must be histologically proven and a different histological type from the original neoplasm treated by the radiation therapy [15]. Our cases meet all of these criteria.

Radiation-induced malignant gliomas (RIMGs) from primary germinomas are considered very rare. We searched 4 published cases from 1960 to 2016, and added our case identified at our report (Table 1) [4–6, 16–18]. Mean age was 11.2 years, and mean interval between initial tumor and RIMGs was 10.8 years. Two cases by You et al. [18] and one case by Nishio et al. [17] received craniospinal irradiation and boost to tumor bed. You et al. reported a case that radiation induced tumor appear at the same location as the initial tumor, so a recurrence was misdiagnosed at first [18]. Kitanaka et al. reported a case of 13-year-old boy whose pineal mass was diminished in size with a diagnostic radiation of 20 Gy. Such a susceptibility to radiation confirmed the diagnosis of germinoma, and another 34 Gy was delivered to the whole brain [16].

**Table 1:** Literature Review of Cases of Radiation-Induced Malignant Gliomas (RIMGs) from Germinomas (1960–2016)

	Author (Ref.)	Sex/Age (years)	Site	Dose (Gy)	CTx	Interval (years)	Second Dx.	Site
1	*You* [18]	M/8	BG/Th	50.4	No	9.5	hGGTm	BG/Th
2	*You* [18]	F/5	SS	49.8	Yes	8.8	GB	Rt. T
3	*Kitanaka* [16]	M/13	Pineal	54	No	7	AA	Lt. T - P
4	*Nishio* [17]	M/18	Pineal	50	No	9.5	GB	Cbll ~ Brain stem
5	*Our case*	M/12	Pineal	50.4	No	19.4	GB	BG/Th ~ Brain stem

*BG* basal ganglia, *Th* thalamus, *hGGTm* high-grade glial tumor, *SS* suprasella, *GB* glioblastoma, *AA* anaplastic astrocytoma

Germinoma is considered as one of the most radiosensitive tumors and are curable by RT alone [1, 11, 19]. Although RT has had the highest priority among treatments on intracranial germinomas, agreement with respect to treatment volume, dose, and use of chemotherapy has not been reached. Ogawa et al. [1] reviewed 126 patients with intracranial germinoma and said that higher total doses were effective in preventing intracranial relapse. Haddock et al. [19] reported whole-brain or craniospinal axis irradiation appears to result in fewer spine and brain failures than dose partial-brain irradiation. But, the risk of developing a secondary brain tumor increases with increasing irradiation dose [20]. There have been many efforts to develop minimum effective radiation dose for germinoma, but the optimal radiation dose and its potential to cause secondary tumor have always been difficult to reach conclusion.

Radiation-induced CNS neoplasms are rare, but the cumulative risk of brain tumor after therapeutic cranial irradiation is reported as up to 2.7% at 15 years [21]. Furthermore, Acharya et al. reported that at 25 years, the cumulative incidence of subsequent malignancy after treatment of germinoma was 6.0% [7]. Although RT has the highest priority among treatments on intracranial germinomas, RT-only therapy has the possibility of second primary tumor.

As an effort to reduce dose of RT has begun after the mid-2000s [22–24] and the prognosis of primary germinoma is relatively good, we will face more to the second primary tumors during long-term follow up. As our case revealed, secondary malignancy is possible even after 20 year follow-up. So, when new lesions are developed, especially long period after initial irradiation, we should doubt the chance of radiation-induced secondary tumors. Proper evaluation for new symptoms should be emphasized to the patients and their family, and the pathologic confirmation of a new lesion is important in planning optimal treatment plan.

We document the development of therapy-associated secondary brain tumors in a patient with germinoma who underwent primary tumor resection and postoperative RT without chemotherapy. RT-only therapy without chemotherapy for germinoma is effective, but radiation-induced secondary tumors are possible during long-term follow up, even about 20 years after primary tumor like in our case. So, Patients with germinoma who had only postoperative RT without chemotherapy should be carefully monitored.

## Abbreviations

GCT: Germ cell tumors
IDH: Isocitrate dehydrogenase
MRI: Magnetic resonance imaging
RIMGs: Radiation-induced malignant gliomas
RT: Radiotherapy
